# Lipopolysaccharide-Induced Middle Ear Inflammation Disrupts the cochlear Intra-Strial Fluid–Blood Barrier through Down-Regulation of Tight Junction Proteins

**DOI:** 10.1371/journal.pone.0122572

**Published:** 2015-03-27

**Authors:** Jinhui Zhang, Songlin Chen, Zhiqiang Hou, Jing Cai, Mingmin Dong, Xiaorui Shi

**Affiliations:** 1 Department of Otolaryngology/Head and Neck Surgery, First Affiliated Hospital of Zhengzhou University, Zhengzhou, Henan, China; 2 Oregon Hearing Research Center, Department of Otolaryngology/Head and Neck Surgery, Oregon Health & Science University, Portland, Oregon, United States of America; Texas A&M University Health Science Center College of Medicine & Baylor Scott and White Health, UNITED STATES

## Abstract

Middle ear infection (or inflammation) is the most common pathological condition that causes fluid to accumulate in the middle ear, disrupting cochlear homeostasis. Lipopolysaccharide, a product of bacteriolysis, activates macrophages and causes release of inflammatory cytokines. Many studies have shown that lipopolysaccharides cause functional and structural changes in the inner ear similar to that of inflammation. However, it is specifically not known how lipopolysaccharides affect the blood-labyrinth barrier in the stria vascularis (intra-strial fluid–blood barrier), nor what the underlying mechanisms are. In this study, we used a cell culture-based *in vitro* model and animal-based *in vivo* model, combined with immunohistochemistry and a vascular leakage assay, to investigate lipopolysaccharide effects on the integrity of the mouse intra-strial fluid–blood barrier. Our results show lipopolysaccharide-induced local infection significantly affects intra-strial fluid–blood barrier component cells. Pericytes and perivascular-resident macrophage-like melanocytes are particularly affected, and the morphological and functional changes in these cells are accompanied by substantial changes in barrier integrity. Significant vascular leakage is found in the lipopolysaccharide treated-animals. Consistent with the findings from the *in vivo* animal model, the permeability of the endothelial cell monolayer to FITC-albumin was significantly higher in the lipopolysaccharide-treated monolayer than in an untreated endothelial cell monolayer. Further study has shown the lipopolysaccharide-induced inflammation to have a major effect on the expression of tight junctions in the blood barrier. Lipopolysaccharide was also shown to cause high frequency hearing loss, corroborated by previous reports from other laboratories. Our findings show lipopolysaccharide-evoked middle ear infection disrupts inner ear fluid balance, and its particular effects on the intra-strial fluid–blood barrier, essential for cochlear homeostasis. The barrier is degraded as the expression of tight junction-associated proteins such as zona occludens 1, occludin, and vascular endothelial cadherin are down-regulated.

## Introduction

Otitis media (OM) is a common inflammatory disease resulting in fluid or effusion accumulation in the middle ear cavity [1]. The cause of OM is believed to be multi-factorial, including both viral and bacterial infection of the middle ear cavity [2]. Pathogens implicated in OM include Streptococcus pneumoniae, Streptococcus, and Haemophilus influenzae [3]. Lipopolysaccharide (LPS) is a cell wall constituent of gram negative bacteria known to activate macrophages, mediating production and release of pro-inflammatory cytokines in the host [4, 5]. The LPS toxin may pass into the inner ear through the oval or round window and cause inflammation in the cochlea, or infection of the middle ear or bone surrounding the inner ear [6].

LPS induces functional and structural changes in the inner ear, including direct damage to sensory hair cells [7]. Disruption of inner ear fluid balance, presenting as increased vascular permeability and endolymphatic drops, is focal to the etiology of LPS-induced inner ear inflammation [8–11]. The vascular leakiness associated with LPS is due to the action of multiple inflammatory cytokines [12, 13]. For example, LPS-induced blood-brain-barrier (BBB) damage is associated with pro- and anti-inflammatory cytokine expression of interleukin-1 (IL-1), IL-2, IL8, toll-like receptor 4 (TLR4), nuclear transcription factor kappa-B (NF-κB), p50, and tumor necrosis factor alpha (TNFα). These cytokines activate matrix metalloproteinases, alter transporter function, and markedly affect vascular permeability [5, 14, 15].

In a previous study, we showed injection of bacterial LPS into the middle ear through the tympanic membrane causes vestibular vascular permeability to significantly increase, leading to edema and entry of serum proteins and inflammatory cells. In particular, LPS was noted to cause striking morphological change in perivascular resident macrophage-like melanocytes (PVM/Ms) in the utricle and saccule of the vestibular system [9].

The intra-strial fluid–blood barrier is highly specialized vascular epithelia structured as polygonal loops in the stria vascularis [16, 17]. The barrier is surrounded by a large number of accessory cells, including pericytes (PCs) and PVM/Ms [17, 18]. Strial capillaries have a minor role in blood flow regulation, but a crucial role is maintaining the endocochlear potential, ion transport, and endolymphatic fluid balance essential for the ear’s sensitivity [19–23]. Similar to the BBB and BRB (blood-retina barrier), the permeability of the strial BLB is largely a function of the tightness of intercellular junctions and transport activity. TJ-associated proteins, including occludin, claudins, zonula occludens (ZO), and adherens-junction proteins, are richly represented in the intra-strial fluid–blood barrier. The junction proteins form a tight physical barrier impermeable to most small and large molecular weight solutes [24, 25]. The PCs and PVM/Ms control TJ protein expression, critical for intra-strial fluid–blood barrier integrity. In this study, we investigated the mechanisms underlying LPS-induced intra-strial fluid–blood barrier leakage in both a cell culture-based *in vitro* model and animal-based *in vivo* model. We demonstrated that LPS alters the intra-strial fluid–blood barrier permeability by disrupting barrier structure, down-regulating expression of the TJ-associated proteins ZO-1, occludin, and vascular endothelial cadherin (ve-cadherin), and promoting transport activity.

## Materials and Methods

### Animals and ethics statement

Male C57BL/6J mice (aged 4–6 weeks, stock number: 000664) and NG2DsRedBAC transgenic mice (stock number: 008241000664) were purchased from Jackson Laboratory (Bar Harbor, ME, USA) and used in this study. The NG2DsRedBAC transgenic mice were backcrossed for more than 20 generations with the C57BL/6 wild-type mice. All procedures were reviewed and approved by the Institutional Animal Care and Use Committee at Oregon Health & Science University (IACUC approval number MU7_IS00001157).

### LPS treatment

*In vivo*, animals in the LPS group were given a trans-tympanic injection into the middle ear with a 10 μl emulsion of LPS in 0.9% sodium chloride (LPS 5 mg/ml, Sigma, USA) 48 hours prior to experimentation. The control group received trans-tympanic injections with 0.9% sodium chloride into the middle ear as previously reported [9]. *In vitro*, primary cell lines were treated with LPS at the concentration of 1 μg/ml. Primary cell lines (EC, PC, and PVM/M) used in these experiments were isolated from the stria vascularis using a method previously reported by our lab [26].

### Auditory testing

An auditory brain-stem response (ABR) audiometry test to pure tones was used to evaluate hearing function. Animals were anesthetized with an injection of ketamine (40 mg/kg) (JHP Phamaceuticals, Rochester, MI) and xylazine (10 mg/kg) (LLOYD Inc., Shenandoah, IA), and placed on a heating pad in a sound-isolated chamber. Needle electrodes were placed subcutaneously near the test ear, at the vertex, and on the contralateral ear. Each ear was stimulated separately with a closed tube sound delivery system sealed into the ear canal. The auditory brain-stem response to a 1-ms rise-time tone burst at 4, 8, 12, 16, 24, and 32 kHz was recorded, and thresholds obtained for each ear. Threshold is defined as an evoked response of 0.2 μV.

### Paraffin embedding

After the mice were sacrificed, cochleae were harvested and fixed in 4% paraformaldehyde overnight at 4°C, rinsed in 37°C PBS (pH 7.3) to remove any residual 4% paraformaldehyde, and decalcified in Decal Overnight Bone Decalcifier (Decal Chemical Corporation, Tallman, NY) overnight. Decalcified cochleae were rinsed free of decalcifier with 2 changes of PBS, and then dehydrated in graded ethanol baths from 70–100%. Tissue was cleared with at least 2 changes of citrisolve until the tissue was fully translucent, and then infiltrated with paraffin wax embedding medium under vacuum at 56°C, with 2 baths of 45 min each. Cochleae were oriented in a tissue mold and embedded in the paraffin wax. Tissue sections 5 μm thick were cut, collected on Superfrost Plus glass slides, and adhered to the slides by incubating them for 2 hours at 60°C. The specimens were deparaffinized with citrisolve and rehydrated using graded ethanol steps, followed by immersion in PBS for 2 min, and viewed under an FV1000 Olympus laser-scanning confocal microscope (Olympus, Tokyo, Japan).

### Immunohistochemistry and fluorescence microscopy

The cochleae were harvested and fixed in 4% paraformaldehyde overnight at 4°C, and then rinsed in 37°C PBS (pH 7.3) to remove any residual 4% paraformaldehyde. Immunohistochemistry was performed as previously described [27]. Tissue samples were permeabilized in 0.5% Triton X-100 (Sigma Aldrich, St. Louis, MO) for 30 min, and immunoblocked for 1 hour with a solution of 10% goat serum (Sigma Aldrich, St. Louis, MO) and 1% bovine serum albumin (Fisher Scientific, Pittsburgh, PA) in 0.02 mol/L PBS. The specimens were incubated overnight at 4°C with the primary antibodies (Table 1) diluted in 1% BSA-PBS. After 3 washes in PBS for 30 min, the samples were incubated with secondary antibodies (Table 1) for 1 hour at room temperature. Capillaries were labeled with lectin Griffonia simplicifolia IB4 (GS-IB4) conjugated to Alexa Fluor 568 (Cat#I21412, Life Technologies, Eugene, OR), or Alexa Fluor 488 (Cat#I21411, Life Technologies, Eugene, OR). The tissues were washed in PBS for 30 min, mounted (H-1000, Vector Laboratories, USA), and visualized under an FV1000 Olympus laser-scanning confocal microscope (Olympus, Tokyo, Japan). Controls were prepared by replacing primary antibodies with overnight incubation in 1% BSA-PBS.

**Table 1:** Antibodies applied.

**Antibodies**	**Vectors**	**Identification**	**Dilution**	**Source**	**Specificity (reacts with)**
ZO-1	Invitrogen, Camarillo, CA	Cat# 61–7300	1:25(with 1% BSA-PBS)	Rabbit polyclonal	Human, mouse
VE-cadherin	Abcam, Cambridge, MA	Cat#Ab33168	1:50(with 1% BSA-PBS)	Rabbit polyclonal	Mouse, human
Occludin	Abcam, Cambridge, MA	Cat#Ab31721	1:50(with 1% BSA-PBS)	Rabbit polyclonal	Mouse, rat, human, pig
Desmin	Abcam, Cambridge, MA	Cat#Ab32362	1:50 (with 1% BSA-PBS)	Rabbit monoclonal	Mouse, rat, guinea pig,
Alpha-Tublin	LI-COR, Lincoln, NE	Cat# 926–42213	1:200(with 1% BSA-PBS)	Mouse monoclonal	Human, mouse, rat, monkey
F4/80	eBioscience, San Diego, CA	Cat# 14-4801-85	1:50 (with 1% BSA-PBS)	Rat monoclonal	Mouse
GAPDH	Santa Cruz Biotechnology, Dallas, TX	Cat# sc-20357	1:200(with 1% BSA-PBS)	Goat polyclonal	Mouse, rat, human
Alexa Fluor 568-conjugated goat anti-rabbit IgG	Invitrogen, Eugene, OR	Cat# A-11011	1:100(with 1% BSA-PBS)	Goat	Rabbit
Alexa Fluor 647-conjugated goat anti-rabbit IgG	Invitrogen, Eugene, OR	Cat# A21244	1:100(with 1% BSA-PBS)	Goat	Rabbit
Alexa Fluor 488-conjugated goat anti-rat IgG	Invitrogen, Eugene, OR	Cat# A-11006	1:100(with 1% BSA-PBS)	Goat	Rat
Alexa Fluor 568-conjugated goat anti-rat IgG	Invitrogen, Eugene, OR	Cat#A-11077	1:100(with 1% BSA-PBS)	Goat	Rat
Peroxidase-labeled anti-mouse antibody	GE Healthcare Bio-Sciences, Pittsburgh,PA	Cat# NIF825	1:25000(with 5% skim milk)	Sheep	Mouse
Peroxidase-labeled anti-rabbit antibody	GE Healthcare Bio-Sciences, Pittsburgh,PA	Cat# NIF824	1: 5000(with 5% skim milk)	Donkey	Rabbit
Donkey anti-goat IgG-HRP	Santa Cruz Biotechnology, Dallas, TX	Cat# Sc-2020	1: 5000(with 5% skim milk)	Donkey	Goat

### Frozen sections and immunolabeling

Cochleae from the control and LPS-treated animals were isolated from young mice, and fixed in 4% PFA overnight. After decalcification in bone decalcifier (Decal Chemical Corporation, Tallman, NY) overnight, the cochleae were dehydrated in 30% sucrose and embedded with OCT. Seven-micrometer sections of the cochleae were cut in the mid-modiolar plane. The crysolides were washed with PBS for 15 min to remove the OCT component, and sections permeabilized in 0.5% Triton X-100 (Sigma Aldrich) for 30 min and incubated in 10% normal goat serum (or other similar serum) diluted in PBS for 1 hour to block nonspecific binding sites. The sections were incubated for 12–16 hours with primary antibody F4/80 (see Table 1) at 4°C. Subsequently, the sections were exposed to an appropriate fluorescence-conjugated secondary antibody (Table 1) and GS-IB4 conjugated to Alexa Fluor 488 at 37°C for 1 hour. The tissues were washed for 30 min, mounted (H-1000, Vector Laboratories, USA), sealed in 20% glycerol, and visualized under an FV1000 Olympus laser-scanning confocal microscope. Controls were prepared by replacing primary antibodies with overnight incubation in 1% BSA-PBS.

### Cell staining

Purified primary cultured PVM/Ms at passage 3 were seeded in a glass well dish, and treated with LPS at 1μg/ml for 48 hours. The PVM/Ms were fixed in 4% PFA (pH 7.2) for 15 min, permeabilized in 0.5% Triton X-100 in PBS-BSA for 3 min, incubated with 10% goat serum, and incubated overnight at 4°C with primary antibody for rat monoclonal F4/80 (eBioscience, San Diego, CA) diluted 1:50 in 1% PBS-bovine serum albumin. After 3 washes in PBS, the samples were incubated with secondary antibodies, Alexa Fluor 568 goat anti-rat IgG (H+L) (Life Technologies, Eugene, OR) or lectin Griffonia simplicifolia IB4 (GS-IB4) conjugated to Alexa Fluor 488 (Life Technologies, Eugene, OR) diluted in 1% PBS-bovine serum albumin at 1:100 for 1 hour at room temperature. The cells were washed in 2 ml PBS (3 times for 10 min) and imaged under an FV1000 Olympus laser-scanning confocal microscope.

Purified PCs transfected with pmOrange2-N1 Vector (Clontech Laboratories, Inc., Mountain View, CA) were seeded in the glass well dish, and treated with LPS at 1 μg/ml for 48 hours, and imaged under an FV1000 Olympus laser-scanning confocal microscope with a standard 559 nm laser excitation line.

### Reverse transcription-polymerase chain reaction (RT-PCR) and real-time quantitative RT-PCR (qRT-PCR)

The procedure used for quantitative real-time PCR was described previously [28]. For the *in vitro* models, total RNA from cultured ECs from different groups was extracted separately using RNeasy (Qiagen, Valencia, CA, USA) per the manufacturer's recommendations. For the *in vivo* models, total RNA from strial capillaries of control and LPS-treated groups was extracted separately using RNeasy (QIAGEN). Each group of 5 mice was analyzed for ZO-1, occludin, ve-cadherin, and Gapgh mRNA with qRT-PCR. The sample for total RNA was reverse transcribed with a RETROscript kit (Invitrogen, USA). CDNA synthesized from total RNA was diluted 10-fold with DNase-free water, each cDNA sample independently measured 3 times. Transcripts were quantitated by gene expression assay (Invitrogen): ZO-1 (Mm00493699_m1), occludin (Mm00500912_m1), and ve-cadherin (Mm03053719_s1) on a model 7300 real-time PCR system (Applied Biosystems, Foster City, CA, USA). The real-time PCR was cycled at 95°C for 20 s, 40 cycles at 95°C for 1 s, and 60°C for 20 s. Mouse Gapdh was the endogenous control. Quantitative PCR was performed per the guidelines provided by Applied Biosystems and analyzed using the comparative cycle threshold method.

### Transmission electron microscopy (TEM)

The temporal bones were isolated, and the cochlea perfused through the round window. This was followed by immersion in a fixative of 4% (wt/vol) paraformaldehyde -0.1% (vol/vol) glutaraldehyde in 0.1 M phosphate-buffer overnight. Stria vascularis tissues were dissected and post fixed in 1% osmium (Electron Microscopy Sciences, Hatfield, PA). Tissues were dehydrated with a graded alcohol series and embedded in Embed 812 (Electron Microscopy Sciences, Hatfield, PA), sectioned, stained with lead citrate (Electron Microscopy Sciences, Hatfield, PA) and uranyl acetate (Electron Microscopy Sciences, Hatfield, PA), and viewed on a Philips CM 100 transmission electron microscope (Philips/FEI Corporation, Eindhoven, Holland).

### Western blot

Total protein (50 μg) from each sample was added to a 10% sodium dodecyl sulfate–polyacrylamide gel to detect ZO-1, occludin, vascular endothelial cadherin (ve-cadherin), and a-tubulin. Proteins were electrophoretically transferred to PVDF membranes and blocked with nonfat milk for 1 hour at room temperature. Specific immunodetection was carried out by incubation with primary antibodies, either anti-ZO-1 antibody diluted 1:50, or anti-occudin 1:100 and anti-ve-cadherin antibody diluted (1:250) in skim milk overnight at 4°C. After 3 washes with TBST, the membranes were incubated for 1 hour with secondary antibody (information on the antibodies can be found in Table 1), and antigens were assessed using ECL plus Western blotting Detection Reagents (Amersham, Arlington Heights, IL, USA).

### In-cell Western blotting

ECs were seeded at the same concentration (1.3×10^5^ cells/ml, 100 μl per well) in a 96-well plate and incubated until the cells consistently adhered to the bottom of the plate. For the LPS group, the cells were incubated with LPS at 1μg/ml for 48 hours. The cells were fixed in 4% formaldehyde for 20 min at room temperature. The cells were permeabilized with 1×PBS containing 0.1% Triton X-100, blocked in blocking buffer (927–40000,LI-COR Biosciences, Lincoln, NE, USA) for 90 min, and incubated with primary antibodies for ZO-1, occludin, ve-cadherin, and α-Tublin (Table 1) for 2 hours. The cells were then washed 4 times for 5 min in PBS containing 0.1% Tween-20 and incubated with 1:200 secondary antibodies including donkey anti-mouse IRDye 800CW (926–32212 LI-COR, USA) and donkey anti-rabbit IRDye 680RD (926–68073, LI-COR, USA) for 1 hour at room temperature. Cells were washed again 3 times in PBS. Stained cells were imaged with an Odyssey Imager (Li-COR Biosciences) and analyzed.

### Immunolabeling of TJ-associated proteins

Purified endothelial cells at passage 3 and at a density of 4×10^4^/cm^2^ were seeded and grown on a glass well dish until an endothelial cell monolayer formed. The cells were incubated with LPS at 1 μg/ml for 24 hours. The cells were then fixed in 4% PFA (pH 7.2) for 15 min at room temperature and washed in 2 ml PBS (3 times for 10 min). The cells were permeabilized in 0.5% Triton X-100 in PBS-BSA for 3 min at room temperature, washed with 2 ml PBS (three times for 10 min), and incubated with an immunofluorescence blocking solution at room temperature. The cells were incubated with primary antibodies (Table 1) diluted in a 1% PBS-BSA solution overnight at 4°C, washed in 2 ml PBS (three times for 10 min), and incubated with secondary antibodies (Table 1) diluted in 1% BSA-PBS solution for 1 hour at room temperature. The cells were washed in 2 ml PBS (3 times for 10 min) and imaged under a confocal laser scanning microscope.

### Assessment of vascular permeability *in vivo*

Vascular permeability in the control and LPS treated group was assessed using a fluorescein isothiocyanate-conjugated bovine albumin tracer (FITC-albumin, 66 kDa, A-9771; Sigma, Cream Ridge, NJ, USA). The tracer was intravenously administered to the tail vein of anesthetized animals 30 min prior to transcardiac perfusion with PBS. The stria vascularis was removed and homogenized in 1% Triton X-100 in PBS, and the lysate centrifuged at 13,000 rpm for 20 min. Relative fluorescence of the supernatant was measured on a TecanGENios Plus microplate reader (Tecan Group Ltd, San Jose, CA, USA).

Vascular permeability *in vivo* was also assessed by recording tracer movement through a specially prepared vessel window, as described previously [29]. Animals were anesthetized and wrapped in a heating pad, with rectal temperature maintained at 38.5°C. A lateral and ventral approach was used to open the left bulla and create a rectangular fenestration (0.1×0.1 mm) in the cochlea at the basal turn. FITC-conjugated to bovine albumin was slowly administrated intravenously to the mouse at a concentration of 40 mg/ml in 0.1 ml physiological solution for 10 min prior to recording.

### Permeability assay

ECs (5×10^5^) were seeded on the insert of a Transwell filter (Coring 3460, 0.4 μm pore size) in 24-well dishes and grown 2–3 days until they reached confluence. After treatment with LPS (1 μg/ml), FITC–dextran (FD10S, Sigma, USA) was added to the upper chamber at a final concentration of 1mg/mL. After 30 min of incubation at 37°C, 50 μL samples were taken from the lower chamber of each Transwell and transferred to a 96-well plate for fluorescence measurements. The fluorescent content of the samples was measured using a fluorescence plate reader optically filtered for 485nm excitation and 535 nm emission [30].

### Statistics

Data, presented as means ± SD, were evaluated using the Student's t-test for comparison of two groups or ANOVA for comparisons of three or more groups. A 95% confidence level was considered statistically significant.

## Results

### Evaluation of the LPS-induced mouse acute otitis media (OM) model

The tympanic membrane, middle ear mucosa, and inflammatory cell infiltration in the middle ear were observed in the LPS-induced OM mouse model.

**Fig 1:**
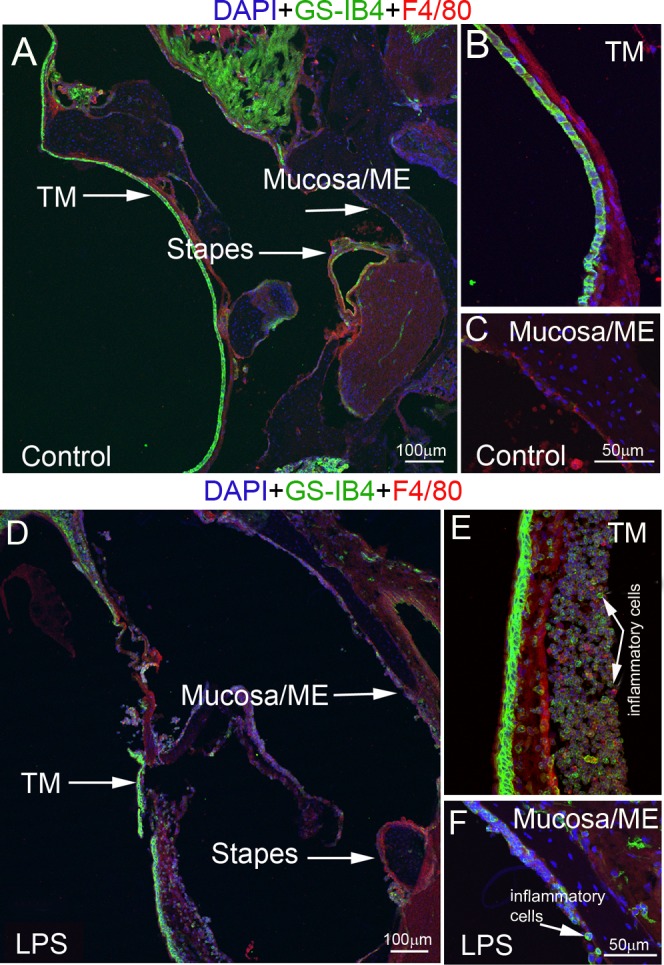
Confocal projection images of the tympanic membrane and mucosa of the middle ear in control and LPS-treated mice. Confocal maximum projection images of the tympanic membrane and mucosa of the middle ear in control (A, B and C) and LPS-treated mice (D, E and F). (A) is a low magnification image showing the normal tympanic membrane (TM) and mucosa in the middle ear (mucosa/ME) of a control animal. (**B**) and (**C**) are high magnification images of the TM and mucosa/ME in a control animal. (**D**) is a low magnification image showing thickening of the TM and swelling of the mucosa/ME typically observed in LPS-treated animals. (**E**) and (**F**) are high magnification images showing a large number of infiltrated cells expressing F4/80 (red, arrow) and positive for GS-IB4 (green, arrow) in the region of the TM and mucosa/SEM in LPS-treated animals. TM = tympanic membrane; ME = middle ear.

Under normal conditions, the middle ear cavity was clear of inflammation (**Fig 1A**). The tympanic membrane and middle ear mucosa were much thinner than in the controls as shown in **Figs 1A**(low magnification) and **1B and 1C**(high magnification). In contrast, the tympanic membrane and mucosa of the middle ear in the LPS-treated ears were much thicker, both showing infiltration of GS-IB4 positive and GS-IB4 negative F4/80 positive inflammatory cells as shown in **Fig 1D**. **Fig 1E and 1F**further display views of the bulkier tympanic membrane and mucosa of the middle ear as well as infiltration of F4/80 positive inflammatory cells in the LPS-treated controls under high magnification.

### LPS affects the structure of the intra-strial fluid–blood barrier

**Fig 2:**
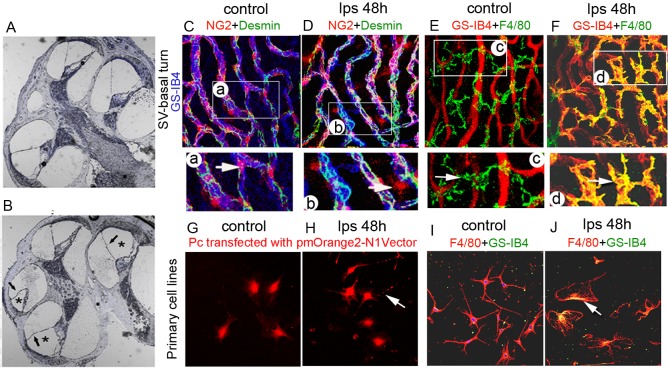
LPS treatment “activates” PCs and PVM/Ms in the intra-strial fluid–blood barrier. LPS treatment “activates” PCs and PVM/Ms in the intra-strial fluid–blood barrier. (**A**) and (**B**) Cochlear cross-sections show slight hydrops in LPS-treated animals (black arrows/stars). (**C**) A representative confocal 3D maximum projection shows normal, inactivated PCs *in vivo* (in transgenic mice with fluorescent labeled NG2, red) to have a flat and slender cell body. The cell morphology is clearly shown in the enlarged image from region of (a) in **Fig 2C**. (**D**) LPS stimulated PCs display a “prominent round” cell body in less physical contact with the strial capillary and release a large number of particles which can be easily seen in the enlarged image from region of (b) in **Fig 2D** (indicated by the arrow). (**E**) PVM/M distribution on strial capillaries, shown labeled with GS-IB4 in a control animal. **Fig E** (c) is an enlarged image from region of (c) in **Fig 2E.** The PVM/M enshrouds a capillary network with its slander dendritic branches (indicated by the arrow). (**F**) PVM/Ms in LPS-treated animals display a reduced amount of branching, as well as withdrawal of ramifications. PVM/Ms in LPS-treated animals are in less physical contact with capillaries. A subset of the PVM/Ms is activated, indicated by display of a terminal galactopyranosyl group on the membrane surface and binding with GS-IB4. **Fig 2F** (d) is an enlarged image from region of (c) in **Fig 2F.** Here the PVM/M is shown still surrounding the capillary network, but with withdrawal of dendritic branches (arrow). **(G)** Primary cell lines of cultured PCs are large, flat, stellate shaped cells, with a broadfilopod morphology. (**H)** LPS treatment for 48 hours causes PCs to branch and release a large number of particles. (**I**) Primary cell lines of cultured PVM/Ms under control conditions display a unique pattern of dendritic processes. GS-IB4 fluorescence in the control (non-LPS treated) cell line was undetectable. (**J**) Some perivascular resident macrophages are activated, displaying a terminal galactopyranosyl group on the membrane surface and binding the lectin Griffonia simplicifolia-IB4 (arrows, GS-IB4). The data show PCs and PVM/Ms are highly responsive to LPS. The control and LPS-treated groups show clear signs of morphological changes.

Compared with non-LPS-treated animal (**Fig 2A**), a slight endolymphatic hydrops was noticed in the LPS-treated animals (**Fig 2B**). In addition to causing hearing defects and slightly abnormal lymphatic volume, LPS also disrupts accessory component cells and causes changes in the structure of the intra-strial fluid–blood barrier.

The primary accessory cells in the intra-strial fluid–blood barrier are PCs and PVM/Ms [18, 27, 31, 32]. PCs and PVM/Ms are essential for maintaining the physical stability and functional integrity of the intra-strial fluid–blood barrier [25]. In this study, we observed how PCs respond to LPS treatment in a transgenic mouse model, with the PCs expressing a bright red fluorescence variant (NG2DsRed.T1) under the control of a mouse NG2 (Cspg4) promoter [33]. Under normal conditions, PCs (NG2 positive) exhibit a flat and slender morphology, tightly associated with strial capillaries (**Fig 2C**). Changed morphology is clearly visualized in the enlarged image from region (a) in **Fig 2C.** Morphological changes are seen in the PCs after 48 hours of LPS treatment. The LPS-treated PCs display a prominent round body, and are found peeling away from the capillary wall, previously described as a sign of PC migration [34]. In addition to the migration, the LPS-induced the PCs to release a great number of unknown particles (**Fig 2D**), shown in an enlarged image (**b**) from **Fig 2D.**

LPS treatment also affects the morphology of PVM/Ms (**Fig 2E and 2F**). Under normal conditions, most PVM/Ms surround capillary networks with long dendritic branches. Normal PVM/Ms do not display terminal galactopyranosyl groups on their membranes, therefore they do not bind to Alexa Fluor 488 conjugated-GS-IB4 (**Fig 2E**). However, after 48 hours LPS treatment, morphology of the PVM/Ms was changed. The PVM/Ms become smaller with shorter processes (**Fig 2F**). Some of them become flat and amoeboid shaped. Most noticeably, the LPS stimulates PVM/Ms to expose their terminal galactopyranosyl group on their membrane surface, binding to Alexa Fluor 488 conjugated-GS-IB4, a hallmark of macrophage activation [35, 36]. Changed morphology and PVM/M activation is clearly visualized in the enlarged image from region (d) in **Fig 2F** compared with normal morphology from region (c) in **Fig 2E.**

We further examined the LPS-induced changes in PCs and PVM/Ms in primary cell lines. Under normal conditions (not LPS stimulated), the cultured cochlear PCs, shown in **Fig 2G** , exhibit as large, flat, stellate shaped cells, with a broad filopod morphology as we previously reported [37], and also described by others [38, 39]. We found that the LPS treatment caused the PCs to release a large number of unknown particles (**Fig 2H**), also seen in our *in vivo* animal model.

PVM/Ms normally display multiple dendritic processes *in vitro* (**Fig 2I**). Consistent with the findings in the *in vivo* animal model, LPS treatment for 48 hours caused some PVM/Ms to activate and display a terminal galactopyranosyl group on the membrane surface which binds to GS-IB4 (**Fig 2J**). GS-IB4 binding was undetectable in control (non-LPS treated) cell lines.

The data show that PCs and PVM/Ms are highly responsive to LPS.

### LPS induces a functional change in the intra-strial fluid–blood barrier *in vivo* and *in vitro*

The intra-strial fluid–blood barrier restricts molecular transport from the blood into the ear, and is essential for maintaining cochlear homeostasis under normal conditions [22]. In this study, we found that the barrier becomes highly permeable to injected serum proteins, such as albumin-FITC with LPS treatment.

**Fig 3:**
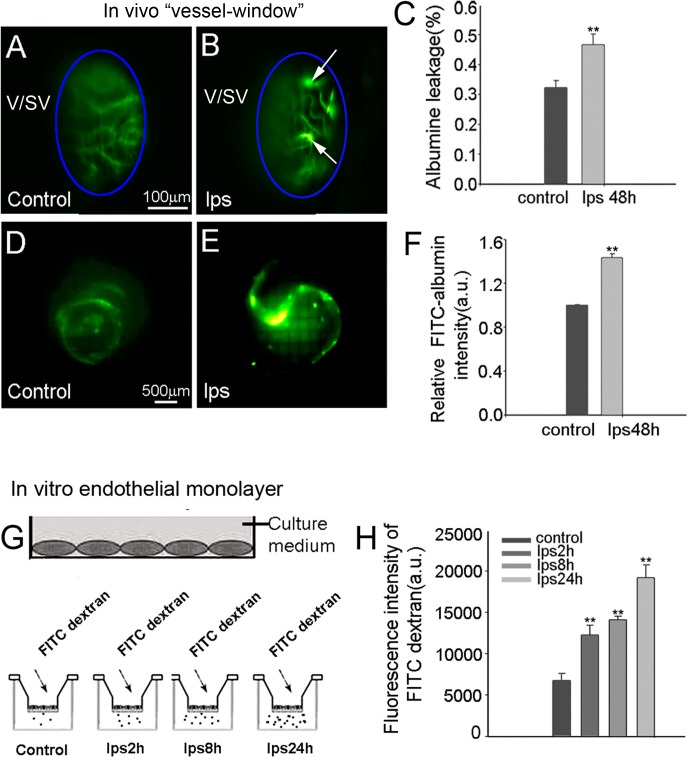
LPS treatment increases vascular leakage. LPS treatment increases vascular leakage. (**A**) Under normal conditions, the FITC-albumin is restricted to the vascular wall of the stria vascularis, and control animals show no sites of obvious leakage. **(B**) In LPS-treated animals, however, capillaries are permeable and multiple sites of vascular leakage are seen in the vessel of the stria vascularis. (**C**) Significantly more leakage of albumin-FITC is seen in LPS-treated animals than in control animals (**P<0.01). (**D**) Retention of FITC-albumin in isolated whole mounts of untreated cochlear stria vascularis is relatively low in non-LPS treated mice, and (**E**) retention in the LPS-treated mice is much stronger. (**F**) FITC-albumin fluorescence is significantly higher in the LPS-treated group than in control animals (**P<0.01). (**G**) and (**H**) The graph shows the effect of LPS on the permeability of tight junctions in an EC monolayer to FITC-dextran. Permeability was measured by determining the flux of FITC-dextran from the upper to the lower chamber of the Transwell filter. Data are expressed as means ± SD (**P<0.01).

In this study, vascular permeability was assessed in both the *in vivo* animal based and *in vitro* EC line based models. In the *in vivo* animal model, vascular permeability in control and LPS-treated animals was assessed using a FITC-conjugated bovine albumin tracer. FITC-albumin was administered intravenously to the mouse at a dose of 40mg/ml in a physiological solution for 10 min prior to imaging. Fluorescence tracer movement through the capillaries was observed and recorded through a prepared vessel-window in the cochlear lateral wall with an intra-vital fluorescence microscope [29]. The FITC-albumin was found restricted to the luminal vascular wall in control animals (**Fig 3A**). With 48 hours of LPS treatment, the capillaries become demonstrably more permeable. **Fig 3B** shows vascular permeability increased at multiple sites in LPS-treated animals. Albumin leakage was quantified using a leakage index defined in previous publications [21, 29]. Vascular leakage was significantly increased in LPS-treated animals, as shown in **Fig 3C** (n = 5; **P<0.01). Retention of the fluorescent dye was assessed in isolated cochlear lateral wall 30 min after administration of FITC-albumin to the animals. We found retained fluorescence in LPS treated animals significantly higher than in non-treated (control) animals in isolated whole mounts of the stria vascularis (see **Fig 3D and 3E**). LPS-treated animals show significant leakage of albumin-FITC across the intra-strial fluid–blood barrier (**Fig 3F,** n = 5;**P<0.01).

The effect of LPS on endothelial monolayer permeability was also assessed as the flux of FITC-dextran across an EC monolayer in an *in vitro* endothelial monolayer model. A primary ECcell line at passage 3 was seeded on Transwell filters for 3–5 days to form a monolayer, and subsequently stimulated with LPS at 1 μg/ml for 2 hours, 8 hours, and 24 hours. Permeability was assessed by determining the flux of FITC-dextran (MW 10kDa) in the upper and lower chambers. Consistent with our *in vivo* finding, we found the endothelial monolayer barrier more permeable in the LPS-treated groups, shown in **Fig 3G and 3H**(n = 6, **P<0.01).

### LPS down-regulates expression of endothelial-endothelial TJ proteins

**Fig 4:**
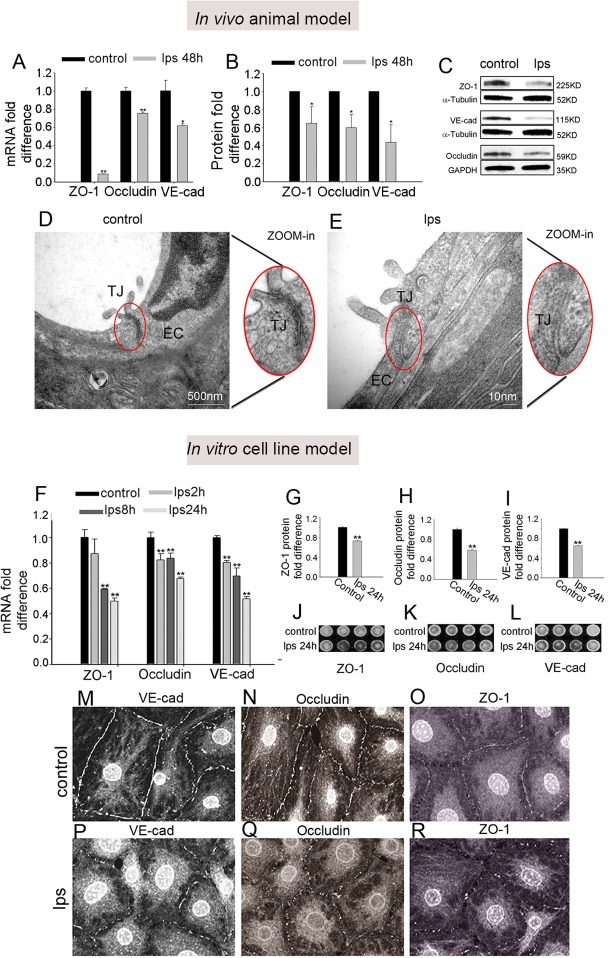
LPS affects the expression of tight-junction proteins. LPS affects the expression of tight-junction proteins. (**A**) qRT-PCR shows mRNA expression for ZO-1, occludin, and ve-cadherin in control and LPS treated animals (**P<0.01, *P <0.05). (**B**) Quantitative analysis of ZO-1, occludin, and ve-cadherin protein expression in control and LPS-treated groups (*P<0.05). (**C**) Western blot analysis shows significantly decreased expression of ZO-1, occludin, and ve-cadherin in the LPS-treated group. (**D**) TEM images show TJ contact points are extensive in control mice (Left, zoomed inset). The left window (zoomed inset) displays a higher magnification of the TJs between ECs. (**E**) Alterations in the TJ ultrastructure are seen in the LPS-treated mice. TJ contact points were reduced in number in response to LPS (left, inset). (**F**) qRT-PCR shows mRNA for ZO-1, occludin, and ve-cadherin in the endothelial monolayer of the control and LPS group at different time points (**P<0.01, *P<0.05). (**G**-**I**) Quantitative analysis of protein expression for ZO-1, occludin, and ve-cadherin in control and LPS-treated groups (**P<0.01). (**J**-**L**) In-cell Western blot analysis of tight junction protein expression. (**M**-**R**) Representative confocal maximum projection images show immunofluorescent labeling of tight junction protein expression, including ZO-1, occludin, and ve-cadherin, in the EC monolayer.

The permeability properties of the intra-strial fluid–blood barrier are largely a function of the tightness of the intercellular junction. The major TJ-associated proteins in the barrier are occludin, claudins, ZO-1, and adherens-junction proteins [40, 41]. Several tight- and adherens-junction proteins, including ZO-1, occludin, and VE-cadherin, have been found in the intra-strial fluid–blood barrier [25, 37]. *In vivo*, mRNA levels for ZO-1, occludin, and ve-cadherin, assessed with quantitative RT-PCR (qRT-PCR), fall dramatically within 48 hours of administering LPS to the ear (**Fig 4A**) (n = 3; **P<0.01). Concurrent with the decreased mRNA expression, protein levels for ZO-1, occludin, and ve-cadherin, analyzed by Western blot, also show a marked decrease (**Fig 4B and 4C)** (n = 3; *P<0.05). Further, TEM images from LPS-treated animals displayed a reduced number of TJ contact points compared to the control mice (**Fig 4D and 4E**), indicating the LPS induced permeability changes are associated with altered TJ organization.

Consistent with the findings from *in vivo* animal model, In the cell line model, mRNA expression for ve-cadherin and occluding in the endothelial monolayer was significantly down regulated at 2 hours, 8 hours, and 24 hours after LPS treatment compared to the control groups, and mRNA for ZO-1 was significantly decreased at 8 hours and 24 hours (**Fig 4F**) (n = 3; **P<0.01). Concurrent with decreased mRNA expression, protein levels for ZO-1, occludin, and ve-cadherin at 24 hours after LPS treatment, analyzed by in-cell western blotting, also showed a marked decrease (**Fig 4G-4L**) (n = 4; **P<0.01). Furthermore, immunohistochemical examination by confocal microscopy clearly showed less staining of TJ proteins at the EC-EC contacts in the LPS-treated group than in the control group (**Fig 4M-4R**).

The consistency between the *in vivo* and *in vitro* results indicates that LPS significantly down-regulated TJ protein expression in the blood barrier.

### LPS causes hearing loss

**Fig 5:**
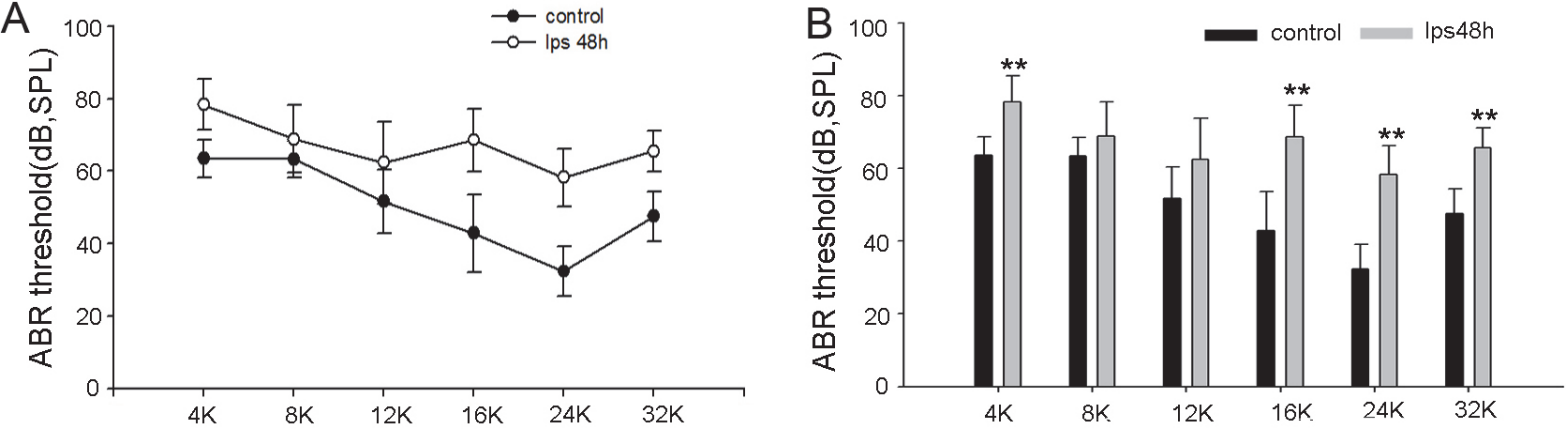
LPS causes hearing loss. LPS damages hearing function. (**A**) and (**B**) show the mean value of hearing threshold levels measured by ABR in control and LPS-treated groups. The ABR threshold in LPS-treated animals is significantly elevated at 4 kHz, 16 kHz, 24 kHz, and 32 kHz (**P<0.01).

Consistent with the findings of another group [42], we found hearing function was affected by LPS treatment. Hearing threshold at most test frequencies was elevated in the LPS-treated group (control group, n = 6; LPS group, n = 10; **P<0.01) (**Fig 5A and 5B**).

## Discussion

In this study, otitis media was simulated by injecting LPS into the middle ear of a mouse for 48 hours. A slight endolymphatic hydrops was observed in the LPS-treated animals. A most significant finding was that LPS strongly affects the structure of the intra-strial fluid–blood barrier by disrupting expression of TJ-associated proteins such as ZO-1, occludin, and ve-cadherin. Consistent with previous reports, we also found that LPS causes hearing loss.

### LPS causes middle ear infection and structural changes in the blood-barrier

Otitis media is a common condition seen in pediatric wards [43]. In this study, otitis media was induced by injecting LPS into the mouse middle ear. Compared with the normal control, LPS-treated animals showed a thicker tympanic membrane and mucosa of the middle ear. A large population of inflammatory cells was found in the vicinity of the tympanic membrane and middle ear mucosa (**Fig 1E and 1F**), indicating an acute otitis media consistent with reports of others [44–46]. LPS-treated animals also showed morphological changes in the intra-strial fluid-blood barrier.

The intra-strial fluid-blood barrier is a specialized vascular structure with a dense population of PCs. PCs, as pluripotent progenitor cells, are vital for vascular integrity, angiogenesis, and tissue fibrogenesis, but are highly responsive to trauma [27, 31, 37]. The intra-strial fluid-blood barrier also contains a number of PVM/Ms in close contact with vessels through cytoplasmic processes [18]. Both PCs and PVM/Ms are essential for control of intra-strial fluid–blood barrier stability and integrity [25]. The intra-strial fluid-blood barrier is formed by ECs and an underlying basement membrane [37]. ECs connect to each other by TJs [47] and form a diffusion barrier which selectively excludes large molecules from entering the ear [48].The intra-strial fluid–blood barrier plays a primary role in maintaining normal ion concentrations in endolymph and periymph.

PVM/Ms and PCs are regulatory cells in the intra-strial fluid-blood barrier, critical for barrier function. They also serve as “sensors” with a major role in maintaining tissue homeostasis, and presenting a first line of immunological defence. PVM/Ms are a structurally and functionally complex hybrid cell type with characteristics of both macrophage and melanocyte. PVM/Ms are highly responsive to a variety of toxic stimuli including ischemia and infection. Previously we demonstrated that PVM/Ms are extremely sensitive to loud sound, and undergo dramatic morphological and functional changes in response to loud sound stimulation. An early study by Gratton [49] also showed marked increased activity in melanin-bearing cells of the inner ear in response to the stress of LPS-induced middle ear infection. In this study, the PVM/Ms are shown to change their morphology and are activated in response to LPS-induced middle ear infection (**Fig 1**). In brain research, activation of astrocytes (or microglia) is a major factor prolonging the release of pro-inflammatory cytokines in the brain [50]. Targeting (suppressing) astrocyte (or microglia) activation attenuates the cytokine release. LPS-triggered activation of PVM/Ms, by its effect on extending cytokine production, could also be extending LPS-induced inner ear inflammation.

PCs, as “the second line of defense” [51], are in close proximity to ECs, and critical for blood barrier integrity [52, 53]. Normally, PCs are embedded in the basement membrane. PC migration, resulting in a break away from the basement membrane and vessels, has been seen in the brain and retina in response to various pathogens [54, 55]. Our previous loud sound study showed the PCs vulnerable and sensitive to the stress of acoustic trauma. Upon exposure to loud sound, PCs migrate from points of normal endothelial attachment [27] and destabilize the BLB in the stria vascularis. Consistent with the PC pathology seen with loud sound stimulation, PCs migrate from the vessel wall and also release a large number of unknown particles when exposed to LPS for 24 hours.

A recent study of PCs in the brain and lung shows the PCs to be a major source of cytokines. They release (secrete) multiple cytokines, including Interleukin (IL)-1 alpha, IL-5, IL-6, Granulocyte-colony stimulating factor (G-CSF), keratinocyte-derived cytokine (KC), monocyte chemotactic protein 1 (MCP1), and RANTES (a member of the IL-8 superfamily of cytokines) when exposed to LPS [56, 57]. PCs are also reported to release transforming growth factor-beta (TGF-β) in response to glycation end-products [58]. It is unknown whether the particles secreted by cochlear PCs on exposure to LPS are cytokines. LPS-induced inflammation in the cochlea, including recruitment of inflammatory neutrophils, was previously reported by different laboratories [7, 59]. Do the particles released by cochlear PCs mediate LPS-induced recruitment of inflammatory neutrophils in the stria vascularis?

The specific signals underlying migration of cochlear PCs and PVM/M activation are unknown, as are the sequelae of the migration and activation. The mechanisms governing PC migration and tissue resident macrophage activation are extremely complex, and multiple signaling pathways, including various cytokines such as vascular endothelial growth factor (VEGF), PDGF-β, and TGF-β are reported to be involved [60–63]. LPS is known to have diverse effects on the inflammatory cascade through activation of Toll-like receptor 4 (TLR-4) [64, 65]. These receptors are expressed in the inner ear by immune cells such as macrophages, and by non-immune cells, including lateral wall fibroblasts and epithelial cells [13]. TLR-4 signaling activates nuclear factor kappa B, the “master switch” for inflammation-mediated cytokine production, and releases mediators such as interleukin-1 (IL-1), IL-2, tumor necrosis factor alpha (TNF-α), and TGF-β, all important cytokines and modulators of the inflammatory response [63, 66]. These receptors might be triggering PC migration and PVM/M activation.

### LPS breaks down the blood barrier by down-regulating expression of TJ proteins

Like in other barrier systems, TJs form the intra-strial fluid–blood barrier which prevents transport into the ear [67]. The major TJ-associated proteins in the barrier are occludin, claudins, ZO-1, and adherens-junction proteins [40, 41]. Several of these tight- and adherens-junction proteins, including ZO-1, occludin, and ve-cadherin have previously been identified in the intra-strial fluid–blood barrier [24, 25]. Each of the proteins plays a unique molecular and regulatory role in maintaining TJ structure and function. Occludin, an integral plasma-membrane protein in the TJs of epithelial cells, is essential for the integrity of the intra-strial fluid-blood barrier [37]. ZO-1, on the other hand, is a recognition protein for TJ placement, and loss of ZO-1 results in disorganization of TJs [68–70]. Ve-cadherin is an endothelial cell specific adhesion molecule located at junctions between ECs which moderates EC contacts and affects the EC through outside-in signaling [71].

In this study, we demonstrated that LPS disrupts the BLB integrity in *in vivo* animal-based and *in vitro* cell line-based models by significantly down-regulating the expression of TJ proteins at both the transcript and protein levels. In support of this evidence, we found less immuno-staining for occludin, ZO-1, and ve-cadherin proteins in EC contacts of the LPS-treated group and loosened TJs between ECs (confirmed by TEM study of the ultra-structure). Further functional studies have shown EC monolayers more permeable with LPS treatment, corroborating the significantly increased BLB vascular permeability seen in LPS-treated animal models.

LPS disruption of TJs could be due to a number of factors: (1) LPS may have a direct detrimental effect on the barrier via cytokines. A number of studies have shown that LPS causes direct damage to the blood barrier by breaking down TJs. This has been shown in organs such as the gut and milk-blood barrier to be mediated by LPS-induced cytokines such as toll-like receptor 4 (TLR4) signaling [64, 72]. (2) LPS may cause damage by inducing PC migration. PCs are extensively branched and tightly embrace the abluminal endothelium wall. PC interaction with the endothelium is vital for vascular development, blood flow regulation, and vascular integrity (vascular permeability) [73–77]. PC pathology leads to vascular dysfunction, seen in many diseases. Evidence is accumulating that PCs control vascular stability by up-regulating TJ protein expression, and by suppressing vesicular transport in the endothelium [78]. PC pathology caused vessel instability and vascular leakage are seen in the brain and other organs [78]. (3) LPS causes damage by inducing PVM/M activation. PVM/Ms in the intra-strial fluid-blood barrier maintain barrier integrity by controlling TJ and adhesive junction protein expression [25]. In particular, in a previous report, we demonstrated that PVM/Ms produce pigment epithelium growth factor (PEDF), a 50-kDa glycoprotein first identified in retinal pigment epithelium cells [21, 25], and essential for stabilizing the intra-strial fluid–blood barrier. PVM/M activation and changes in PVM/M phenotype leads to decreased production of PEDF, and to blood barrier breakdown and tissue edema.

### LPS effects on hearing function

Normal function of the intra-strial fluid-blood barrier requires blood barrier integrity, including intact TJs in the endothelial layer [72]. Breakdown of the EC-EC junction leads to vascular leakage (**Fig 3**). Consistent with earlier reports [79–81], our data showed an elevated hearing threshold 48 hours after LPS was administered to the middle ear. A hearing loss of ~30 dB-SPL in high frequencies is seen relative to controls.

Several factors may be contributing to the hearing loss. (1) LPS may be directly damaging the sensory hair cells. LPS, a product of bacteriolysis, has been demonstrated to damage inner and outer hair cell bundles and to cause the tectorial membrane to swell [7]. (2) LPS has been shown to induce increased cytokine production and reactive free radical damage. Increased cytokine production by LPS in the cochlea was previously demonstrated by Juhn et al. [13] and Quintanilla-Dieck et al. [8]. LPS-induced production of free radicals such as nitric oxide was reported as well by our lab and by other labs [82–84]. In particular, LPS causes inducible nitric oxide synthase (iNOS) expression in various cochlear cells, including inner and outer hair cells, pillar cells, hair cell nerve fibers, and marginal cells. (3) LPS may cause a deficiency in cochlear blood flow. Transduction of sound is metabolically demanding, and normal function of the microvasculature in the lateral wall is critical for maintaining ion transport and fluid balance [85–88]. Disruption of the blood-barrier in the stria vascularis causes a shortage of nutrients and oxygen in the tissue and creates a “toxic” environment with accumulation of harmful metabolites. In addition, leakage of blood-barrier components into the lateral wall of the cochlea with increased blood-barrier permeability may be related to sensorineural hearing loss. Juhn et al. [13] suggested the leakage may hinder K+ recycling through the lateral wall, causing an intra-strial electric shunt which disrupts the endocochlear potential EP as reported by Cohen-Salmon et al. [89].

## Conclusions

In summary, our data show that LPS not only induces middle ear infection, but also causes structural changes in the intra-strial fluid–blood barrier. LPS is shown to have strong effects on PCs and PVM/Ms in the barrier, causing PCs to migrate and release particles, and activation of PVM/Ms. LPS also causes a significant down-regulation of TJ-associated protein expression. Down-regulation of ZO-1, occludin, and ve-cadherin weaken the intra-strial fluid-blood barrier and subsequently leads to blood barrier leakage. The exact mechanisms, however, underlying PC migration and secretion of particles and PVM/M activation are yet to be determined.
